# Quantum of Stress Hyperglycemia at the Time of Initial Diagnosis of Tuberculosis

**DOI:** 10.7759/cureus.36382

**Published:** 2023-03-20

**Authors:** Divya Tarachandani, Kritika Singhal, Abhishek Goyal, Ankur Joshi, Rajnish Joshi

**Affiliations:** 1 Internal Medicine, All India Institute of Medical Sciences, Jodhpur, Jodhpur, IND; 2 Internal Medicine, All India Institute of Medical Sciences, Bhopal, Bhopal, IND; 3 Community and Family Medicine, All India Institute of Medical Sciences, Bhopal, Bhopal, IND; 4 Pulmonary Medicine and Tuberculosis, All India Institute of Medical Sciences, Bhopal, Bhopal, IND

**Keywords:** diabetes mellitus type 2, glycosylated hemoglobin (hba1c), tuberculosis(tb), active pulmonary tuberculosis, stress hyperglycemia

## Abstract

Background

India has a high burden of both tuberculosis (TB) and diabetes mellitus (DM). The National TB Elimination Program recommends testing for glycemic status at the initiation of therapy; however, some individuals with elevated sugar levels might have stress hyperglycemia (SH) instead of true DM. Our aim was to perform a longitudinal glycemic assessment of individuals with TB to identify those with true newly diagnosed DM and those with SH.

Methods

We identified newly diagnosed adults with TB and abstracted information about demography, co-morbidities, disease severity, and glucose-lowering agents. A glycemic assessment was performed at baseline and at the end of six months.

Results

We included 150 patients with TB, and based on their initial HbA1c values, 82 (54.6%, 95% CI: 46-62%) had dysglycemia (30% had HbA1c levels above 6.4% and 24.6% had values between 5.9% and 6.4%) and 31 (20.7%, 95% CI: 14-18%) had SH. Among individuals with both baseline and follow-up glycemic values, 30% of the individuals previously defined as DM were characterized as SH. The proportion of true SH was 43% (95% CI: 33-60%).

Conclusion

Most individuals who have dysglycemia at the time of their TB diagnosis have SH. A close follow-up of such individuals will identify those who really require long-term glucose-lowering therapy.

## Introduction

India carries the greatest burden of tuberculosis (TB) in the world, with an estimated annual incidence of 2.8 million [1-2]. Diabetes mellitus (DM) is the most common of all immunosuppressive conditions that constitute risk factors for active TB. Approximately 12% of all Indians are estimated to be diabetic, i.e., diabetes affects about 70 million individuals [3]. DM increases the likelihood of developing TB by approximately two to three times and is known to cause poor treatment outcomes. Among individuals who have TB, those with DM are twice more likely to suffer mortality or relapse as compared to those without it [1].

The diagnosis of new-onset DM in individuals detected with TB is complex. An instance of dysglycemia concurrent with TB is likely to represent stress hyperglycemia (SH), which is a transient hyperglycemic state arisen in response to an infection [4]. In the setting of an acute infection, an elevated fasting or post-prandial plasma glucose level or worsening of a previously controlled DM suggests SH. The American Diabetes Association (ADA) recommends using glycosylated hemoglobin (HbA1c) levels to distinguish between pre-existing DM and SH. As per the ADA definition, an HbA1c value ≥6.5% indicates pre-existing unrecognized diabetes, whereas an HbA1c value <6.5% indicates SH [5]. However, TB is a disease with a chronic infection, and therefore, it is likely that the SH in TB persists long enough to cause HbA1c elevation, unlike in various acute infections.

In previous studies, 8% to 87% of patients with TB were reported to have hyperglycemia [4,6-9]. Previous studies did not estimate the proportion of SH among patients with newly diagnosed TB [10]. Various studies from Iran [11], Pakistan [12], and Tanzania [6] have, however, reported that more than half of patients with TB and newly diagnosed hyperglycemia achieved normoglycemia after three to six months of TB treatment [13]. Regardless of its cause, hyperglycemia needs to be treated. Whereas short-term treatment suffices in cases of SH, patients with DM require lifelong glucose-lowering therapy.

The aim of this study was to understand HbA1c-based glycemic patterns among patients with TB at the initial diagnosis and after six months and determine the proportion of individuals with newly diagnosed DM (persistence of hyperglycemia) and those who have SH (resolution of hyperglycemia) among individuals with TB.

## Materials and methods

Design and ethics statement

We performed a prospective longitudinal observational study among patients with newly diagnosed TB. The study protocol was approved by the Institute of Human Ethics Committee (IHEC) (Ref LOP/IHECPGRMD016). Written informed consent was sought from all study participants prior to inclusion in the study, and only consenting participants were included.

Setting

The study was conducted at the All India Institute of Medical Sciences, Bhopal, a tertiary care teaching hospital in Central India. Adults with suspected TB were evaluated in the medicine or pulmonary medicine outpatient departments. Diagnostic work-up included microbiologic (demonstration of acid-fast bacilli or nucleic acid amplification tests), pathologic (demonstration of caseating granuloma), imaging (chest radiographs and appropriate CT scans or MRI films), and biochemical evaluation of body fluids. All patients were registered with the Revised National TB Control Program (RNTCP) for treatment and follow-up. Initial glycemic assessment, regular follow-up, and monitoring for glucose-lowering therapies in TB patients are part of standard care.

Participants

The study participants were adults above the age of 18 who were newly diagnosed with pulmonary or extrapulmonary TB based on NTEP guidelines. The individuals who denied consent and had received anti-tubercular drug therapy for one week or longer, were HIV-positive, or were pregnant, were excluded.

Sample size

The sample size was calculated from the key document of the National Framework for Joint TB-Diabetes released by RNTCP-NPCDCS in collaboration with the Directorate General of Health Services, Ministry of Health & Family Welfare, Government of India. This document stated that about 10% of TB cases globally are linked to diabetes. Assuming the difference between the sensitivity of the gold standard (HbA1c) and blood sugar [H0 and H1 (effect size)] to the extent of 0.20, in which we presume that the blood sugar values will be able to capture at least 50% of co-morbid patients, a sample size of 200 was proposed (in which 20 people would be TB-DM co-morbid), which would assign the study 81% power. Here, we were able to have 20 previous TB-DM comorbid patients within the initial 150 of our included participants. Hence, we closed the data collection at 150 participants (of whom >20 were TB-DM co-morbid, old and new included).

Procedure

We performed a structured interview at baseline for demographics, comorbidities, and TB-related variables. Furthermore, information about the glycemic status (fasting plasma glucose, FPG; post-meal plasma glucose, PPG; and HbA1c values) was collected from hospital records. Also, information related to glucose-lowering therapies initiated by their treating physicians was collected at baseline and follow-up. A follow-up was conducted at six months to evaluate the outcome of anti-TB drug therapy and the assessment of HbA1c-based glycemic control.

Definitions

TB was classified as pulmonary if it was confined to the lungs and extra-pulmonary if it was confined to either lymph nodes, pleura, pericardium, abdomen, nervous system, or bones. The occurrence of extensive TB disease in multiple organs was classified as disseminated. Cases that were based on microscopic or molecular confirmation were identified separately from those with a pathologic or imaging-based diagnosis. We classified individuals based on their baseline HbA1c levels as normal (less than 5.9%), impaired glucose tolerance (IGT) (5.9-6.4%), and diabetes (DM) (more than 6.5%) according to ADA definitions. At the baseline, individuals who had elevated fasting and post-meal plasma glucose values but HbA1c levels less than 6.5% were defined as having SH.

On follow-up, the glycemic status was reclassified. Individuals who had the reversion of their glycemic status on follow-up from DM, SH, or IGT to normal were reclassified as having true SH. Individuals initially classified as having DM were classified in the same way if their follow-up HbA1c levels were above or equal to 6.5% or if they were on glucose-lowering therapies at a six-month follow-up. Individuals previously classified as either normal, IGT, or SH with an HbA1c level above 6.5% at follow-up were classified as having new-onset DM. Treatment outcomes were defined as cured, treatment complete, treatment success, treatment failure, and death as per RNTCP definitions.

Statistical analyses

A descriptive statistical analysis of key demographic, clinical, and TB-related variables across glycemic categories (normal, impaired, diabetes, and stress hyperglycemia) was performed. The quantum of stress hyperglycemia was assessed based on standard definitions at baseline and on reclassification at follow-up. A univariate analysis was performed using the chi-square and Fisher’s exact tests for ordinal variables and ANOVA or non-parametric equivalents for continuous variables. Exploratory visualizations like alluvial diagrams, rain, and raincloud plots were created to understand the pathways and distribution from a longitudinal perspective. For all comparisons, a p-value of less than 0.05 was considered statistically significant. All statistical analyses were performed using R version 4.1.2 and the ggalluvial and ggplot2 packages available in the open domain.

## Results

A total of 150 patients with TB were included in this study, which was conducted between March 2020 and September 2021. Ninety-seven participants (64.6%) were males, and the mean age of the participants was 41.68 ± 17.6 years. According to baseline HbA1c values, 68 (45.3%, 95% CI: 37-53%) patients had normoglycemia, and the remaining 82 (54.7%, 95% CI: 46-62%) patients had dysglycemia (HbA1c values compatible with either IGT (n = 37; 24.6%, 95% CI: 18-32%) or DM (n = 45; 30%, 95% CI: 22-38%). Compared to individuals with normoglycemia, those with an HbA1c of greater than 6.5% were more likely to have pulmonary TB, a history of smoking, and previously known DM. Individuals with HbA1c in the IGT range were older, and they had a higher proportion of smokers (Table 1).

**Table 1:** Baseline characteristics of patients with TB (n=150) TB: tuberculosis, DM: diabetes mellitus, BMI: body mass index, SBP: systolic blood pressure, RHZE: rifampicin, isoniazid, pyrazinamide, ethambutol, LES: levofloxacin, ethambutol, streptomycin, ME: moxifloxacin, ethambutol, CBNAAT: cartridge-based nucleic acid amplification test. *These variables are continuous, and one-way ANOVA was performed to evaluate differences across groups. aFisher exact test used for evaluating differences across these variables. The remaining variables were evaluated using the chi-square test.

		HbA1c categories			p-value
		<5.9% (a)	5.9 to 6.4% (b)	>6.5% (c)	
No. of patients n(%)		68 (45.33%)	37 (24.67%)	45 (30%)	
Gender n(%)^a^	Male	35 (36.08%)	27 (27.83%)	35 (36.08%)	0.008
	Female	33 (62.26%)	10 (18.87%)	10 (18.87%)	
Age (SD)*		36.41 ± 17.64	42.95 ± 18.11	48.60 ± 14.60	0.009
Mean years of education (SD)*		10.97 ± 7.16	9.3 ± 5.93	9.47 ± 6.27	0.023
TB location n(%)	Extrapulmonary	40 (55.56%)	18 (25%)	14 (19.44%)	0.015
	Pulmonary	28 (35.90%)	19 (24.36%)	31 (39.74%)	
Diagnosis n(%)^a^	Smear	16 (51.61%)	6 (19.35%)	9 (29.03%)	0.671
	CBNAAT	23 (40.35%)	11 (19.30%)	23 (40.35%)	0.088
	Pathological	20 (50%)	13 (32.50%)	7 (17.50%)	0.108
	Imaging	65 (45.45%)	34 (23.78%)	44 (30.77%)	0.449
	Biochemical	26 (54.16%)	15 (31.25%)	7 (14.58%)	0.018
Risk Factors n (%)	Smoking	6 (16.67%)	13 (36.11%)	17 (47.22%)	0.000
	Known DM^ a^	1 (4.76%)	0	20 (95.24%)	0.000
	Alcohol dependence	16 (39.02%)	9 (21.95%)	16 (39.02%)	0.334
	Prior steroid use^a^	6 (75.00%)	0	2 (25.00%)	0.181
Mean BMI (SD)*		17.52 ± 3.59	17.68 ± 3.81	19.53 ± 4.16	0.104
Mean SBP (SD)*		109.87 ± 19.43	113.89 ± 15.58	117.89 ± 14.98	0.154
Anti-TB drug regimen (first line) n(%)^a^	RHZE	63 (44.68%)	35 (24.82%)	43 (30.50%)	1.000
	LES	4 (50.00%)	2 (25.00%)	2 (25.00%)	
	ME	1 (100.00%)	0	0	
Anti-TB drug regimen (second line) n(%)^a^		6 (66.67%)	1 (11.11%)	2 (22.22%)	0.502
Anti-TB + Steroids n(%)		16 (57.14%)	5 (17.86%)	7 (25.00%)	0.369
Severity of pulmonary tuberculosis n(%)^a^	<33% of lung fields	10 (43.48%)	5 (21.74%)	8 (34.78%)	0.620
	33-66% of lung fields	15 (35.71%)	11 (26.19%)	16 (38.10%)	
	≥66% of lung fields	16 (51.61%)	4 (12.90%)	11 (35.48%)	
Severity of tuberculous meningitis n(%)^a^	Grade I	3 (75.00%)	0	1 (25.00%)	0.527
	Grade II	5 (71.43%)	2 (28.57%)	0	
	Grade III	3 (42.86%)	2 (28.57%)	2 (28.57%)	

Among 68 individuals with an HbA1c value <5.9%, 39 (57.4%) had normoglycemia and showed FPG <100 mg/dL and PPG <140 mg/dL. The remaining 29 (42.6%) had SH, as their FPG or PPG values were above 100 mg/dL and 140 mg/dL, respectively. Among the 37 individuals who had HbA1c values between 5.9% and 6.4%, two had SH as their FPG or PPG values above 126 mg/dL and 200 mg/dL, respectively. Thus, based on standard definitions, at the baseline, 31 (20.7%, 95% CI: 14-18%) individuals had SH.

**Figure 1:**
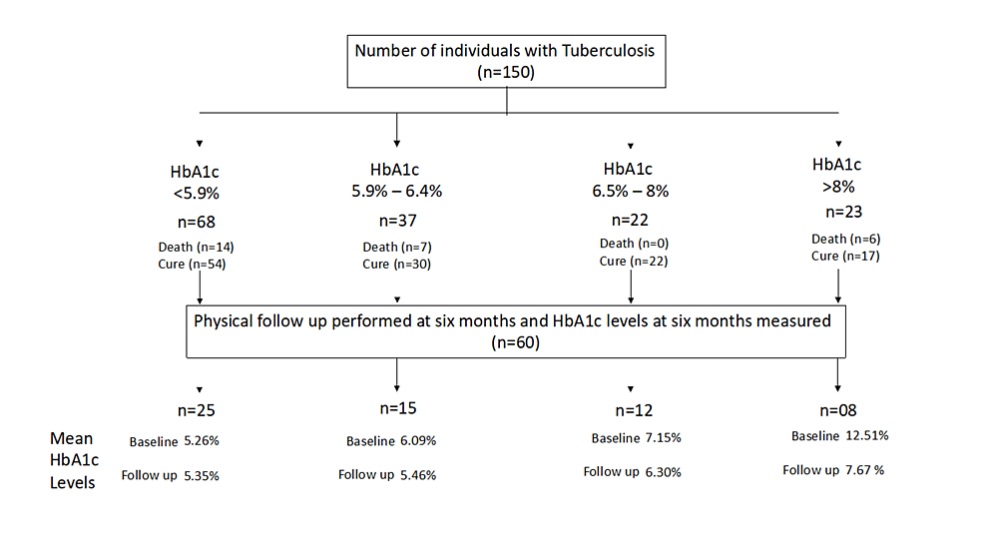
Study flow HbA1c: glycosylated hemoglobin

A total of 27 out of 150 (18%) participants had died prior to the completion of the required six-month follow-up period. The remaining 123 participants (72%) were classified as cured as per available records. We were able to obtain records of HbA1c values for 60 (48.7%) of them. The proportion of individuals at baseline and follow-up was similar across different glycemic groups (Figure 1).

**Figure 2:**
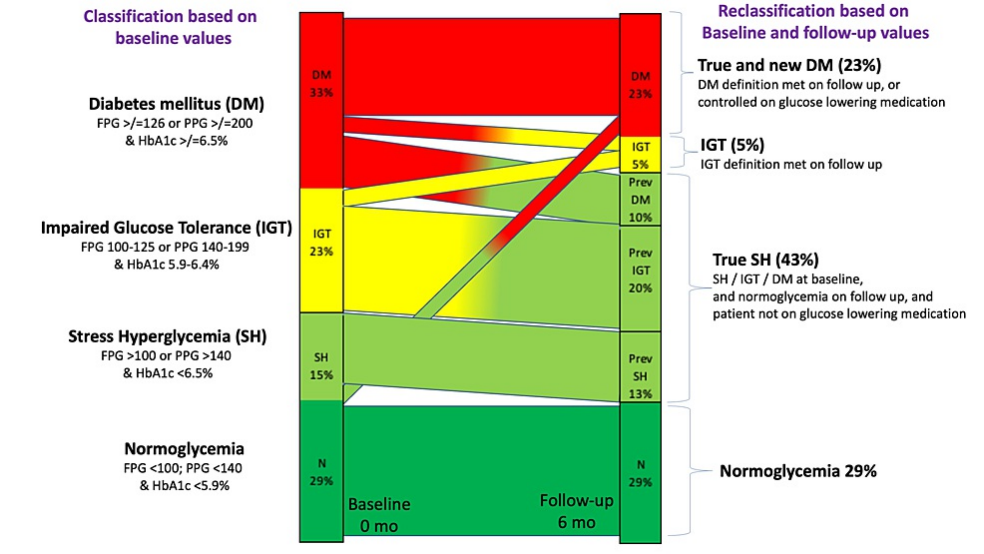
Quantum of stress hyperglycemia based on follow-up reclassification (n=60) All the normoglycemic participants (29%) continued to be in normoglycemic class after 6 months of follow up. A small portion of stress hypoglycemics (2%) converted to true diabetes while remaining stayed as former (13%). Most of the impaired glucose tolerance patients reclassified to stress hypoglycemia (20%) while the rest converted to true diabetics (3%). Further, out of 33% of true diabetics 10% reclassified as stress hyperglycemia and 2% converted to impaired glucose tolerance. In total 43% were classified to be stress hyperglycemic as compared to 15% initially (DM: diabetes mellitus, IGT: impaired glucose tolerance, SH: stress hyperglycemia, true SH: true stress hyperglycemia).

**Figure 3:**
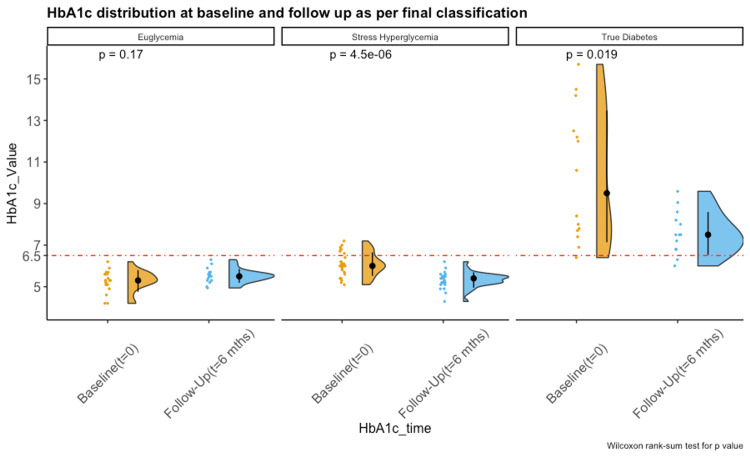
HbA1c distribution at baseline and follow up Above rain-cloud diagram depicts distribution of HbA1c values as per reclassified glycemic status. Red-dashed line shows HbA1c of 6.5%. Baseline distribution is in orange and follow up in blue. Black dot represents mean HbA1c value, and the line depicts its standard errors (HbA1c: glycosylated hemoglobin).

We reclassified the glycemic status of 60 individuals for whom both baseline and follow-up HbA1c values were available. At the baseline, 20 (33%, 95% CI: 21-46%) were classified as DM, 14 (23%, 95% CI: 13-36%) as IGT, 9 (15%, 95% CI: 7-26%) as SH, and 17 (29%, 95% CI: 17-41%) as normoglycemic. On reclassification based on follow-up values, 12 out of 14 (85%) individuals previously classified as IGT and 6 out of 20 (30%) individuals previously classified as DM had true SH. Their FPG, PPG, and HbA1c values on follow-up were in the normoglycemic range without a need for glucose-lowering medications. Only one person progressed from SH at the baseline to DM at the end of treatment at six months. Thus, the proportion of true-SH was 43% (95% CI: 33-60%) and not 15% as estimated at baseline (Figure 2). There was a decline in mean HbA1c values in all groups from baseline to follow-up. Figure 3 shows a declining trend of HbA1c values amid all subgroups classified as per HbA1c.

Participants were reclassified as normal, SH, and DM at the end of six months. While individuals with DM were significantly older, those with true-SH were no different from normoglycemic individuals with respect to demographics, clinical, and TB-related variables (Table 2).

**Table 2:** Characteristics of participants with reclassification based on follow-up values (n=58) TB: tuberculosis, DM: diabetes mellitus, BMI: body mass index, SBP: systolic blood pressure, RHZE: rifampicin, isoniazid, pyrazinamide, ethambutol, LES: levofloxacin, ethambutol, streptomycin, ME: moxifloxacin, ethambutol. *These variables are continuous, and one-way ANOVA was performed to evaluate differences across groups. ^a^Fisher exact test used for evaluating differences across the groups. *Three individuals were classified as having an impaired glucose tolerance and are omitted from this table. ^¶^Normal at baseline and on follow-up. ^#^Stress hyperglycaemia/impaired glucose tolerance (IGT)/DM at baseline but the normal glycemic status on follow-up and without glucose-lowering medication. ^$^Glycemic values compatible with diabetes mellitus at follow-up or person is on glucose-lowering medication.

		Final classification			p-value
		Normal	Stress hyperglycemia#	Diabetes mellitus	
No. of participants		17 (29.31%)	27 (46.55%)	14 (24.14%)	
Gender n(%)^a^	Male	8 (21.05%)	18 (47.37%)	12 (31.58%)	0.078
	Female	9 (45%)	9 (45%)	2 (10%)	
Age (SD)*		26.88 ± 10.13	34.59 ± 15.70	52.50 ± 15.09	0.052
Mean years of schooling		12.94 ± 6.93	11.26 ± 5.60	11 ± 6.78	0.805
Tuberculosis location n(%)^a^	Extrapulmonary	10 (35.71%)	14 (50%)	4 (14.29%)	0.215
	Pulmonary	7 (23.33%)	13 (43.33%)	10 (33.33%)	
Risk factors n (%)^a^	Smoking	2 (16.67%)	6 (50%)	4 (33.33%)	0.497
	Known DM	0	1 (12.50%)	7 (87.50%)	0.000
	Alcohol dependence	4 (25.00%)	7 (43.75%)	5 (31.25%)	0.802
	Prior steroid use	2 (66.67%)	1 (33.33%)	0	0.439
Mean BMI (SD)*		17.75 ± 3.45	18.21 ± 3.24	20.94 ± 4.15	0.532
Mean SBP (SD)*		106.41 ± 12.36	114.22 ± 21.69	122.79 ± 14.78	0.008
Anti-TB drug regimen (first line) n(%)^a^	RHZE	15 (28.85%)	23 (44.23%)	14 (26.92%)	0.310
	LES	1 (20.00%)	4 (80.00%)	0	
	ME	1 (100.00%)	0	0	
Anti-TB drug regimen (second line) n(%)^a^		1 (100.00%)	0	0	0.534
Anti-TB + Steroids n(%)^a^		3 (33.33%)	6 (66.67%)	0	0.165
Severity of pulmonary tuberculosis n(%)^a^	<33% of lung fields	1 (11.11%)	5 (55.56%)	3 (33.33%)	0.559
	33-66% of lung fields	7 (35.00%)	9 (45.00%)	4 (20.00%)	
	≥66% of lung fields	2 (28.57%)	2 (28.57%)	3 (42.86%)	
Severity of tuberculous meningitis n(%)^a^	Grade I	0	1 (100.00%)	0	0.750
	Grade II	1 (33.33%)	2 (66.67%)	0	
	Grade III	0	0	0	

## Discussion

In the current study, we have found that, based on traditional definitions, 15-20% of all patients with TB had SH, 23-25% had impaired glucose tolerance, and 30-33% had DM at the beginning of TB treatment. However, approximately 85% of individuals with impaired glucose tolerance and 30% of all individuals with new-onset DM had normal glycemic levels, without requiring long-term anti-diabetic drug therapy. We estimated the true burden of stress hyperglycemia at 43%.

Hyperglycemia during stress is an evolutionary survival response of the body to severe infections. It aids in metabolic, cardiovascular, and immune responses and is a consequence of increased hepatic gluconeogenesis. This phenomenon is hypothesized to enable the body to meet the increased requirements of activated macrophages [14]. It is especially important in the context of TB, where macrophages are pivotal to containing and controlling the infection. Mild-to-moderate hyperglycemia is also reported to contribute to conferring protection against anti-apoptotic pathways and promoting angiogenesis in ischemic cells due to tubercular infections. Even in critical care settings, hyperglycemia is treated with a higher glycemic threshold. Tight control of this reactionary hyperglycemia could be detrimental [15]; however, long, persisting severe hyperglycemia may produce a counterproductive prothrombotic, proinflammatory, and pro-oxidant effect [16]. Due to these concerns, a high prevalence of DM among patients with TB (inferred from baseline hyperglycemia) is concerning because such individuals are likely to have a higher bacillary load, a greater likelihood of a cavitary lesion, and a higher mortality. Individuals with TB-DM comorbidity not only have heightened levels of pro-inflammatory cytokines prior to the onset of drug therapy, but they also maintain this state through the course of TB treatment [17].

SH can be viewed as increased glycemic variability (GV) in response to alterations in the neuro-chemical axis during inflammatory conditions. From a mathematical perspective, individuals with TB have a high GV at baseline, which reduces over time. Individuals with true DM are likely to have a higher GV as compared to individuals with SH [18]. The decline of inflammation during TB treatment would lead to a concomitant decrease in GV in all individuals. In individuals with SH, GV is expected to normalize, while in DM, it is expected to persist. This physiological phenomenon can best be demonstrated using continuous glucose assessment. We could not find any studies that identify the temporal tipping point for this change in patients with TB. In the absence of robust evidence about the timing of the resolution of SH, the only option for TB control programs is to adapt existing diagnostics to their best abilities [19].

Screening for DM in patients diagnosed with TB is considered an opportunity to identify missed cases [20]. With the launch of the WHO and International Union against TB and Lung Disease collaborative framework for care and control of diabetes and TB in 2011, it was suggested that countries with a high dual burden of the two diseases should formulate techniques to screen for each disease and manage detected cases within routine health services, considering the resources of their setups [21]. Several strategies to cope with the TB-DM challenge have been adopted by the National TB Program with cross-referral to the national chronic disease program (called NPCDCS) [22]. However, there are several issues that need to be addressed. Because a diagnosis of DM is followed by lifelong therapy, diagnostic accuracy is necessary. The diagnostic accuracy of hyperglycemia concomitant with TB is compromised, as the infection is well-known to induce hyperglycemia. Second, the TB control program in India determines glycemic status by capillary blood sugar estimation at a single point in time. Our study demonstrates that dysglycemia is a dynamic entity, and initial diagnostic categorization toward the later part of TB treatment needs revision. There is a need to improve TB-DM algorithms to account for the effects of infection-induced hyperglycemia [23].

There are programmatic challenges with various diagnostic methods for dysglycemia. The oral glucose tolerance test (OGTT) is the gold standard for the diagnosis of DM, especially in young and early diseases. However, the OGTT is difficult to use in programmatic settings due to its cumbersome nature and requirement of multiple blood samples. Other validated methods of diagnosis include FPG, PPG, and random blood sugar (RBS). FPG and PPG require two samples and are well-established in clinical practice. While HbA1c is considered an improvement, our study suggests that in the presence of chronic inflammation, there is a risk of a false-positive diagnosis of DM with single-point testing. A recent study from India demonstrated that HbA1c was better than FPG with an AUC of 0.754 (0.682-0.828) for newly diagnosed DM among patients with TB [24]. Studies from Pakistan and China also report a higher false-positive rate for the diagnosis of DM with FPG than with HbA1c [25-26]. While HbA1c is considered better as compared to RBS and FPG, it has not yet been employed in TB programs [27-28]. Despite conflicting evidence, limitations, and compromised accuracy, RBS is used in the TB control program in India due to convenience [20]. The treating physicians should be aware of the flaws associated with such screenings. SH and the absence of any RBS-defined cut-offs are the most worrying concerns. SH could lead to overdiagnosis of DM, and erroneous higher cut-offs could lead to underdiagnosis.

Our study had certain limitations. First, it was a hospital-based study, so it cannot be generalized to the general population. Second, the ongoing SARS-COVID pandemic led to a reduction in participants at follow-up. There was some loss to follow-up due to death from TB infection. Further, the lack of availability of HbA1c levels at our facility also reduced the number of participants at the follow-up. Despite these limitations, our study describes shortcomings associated with the interpretation of blood glucose levels and a careful primary care approach, including close follow-up of individuals diagnosed with hyperglycemia. Similar studies can be planned in a larger population to understand the trends of hyperglycemia in patients with TB infection.

Our usual practice, adapted from critical care settings, is to initiate therapy for new-onset hyperglycemia in a setting of acute infection above a threshold of 200 mg/dl. We usually repeat the glycemic assessment a week later for all those below this threshold; however, such approaches need to be validated. We suggest that during the course of TB therapy, a close follow-up is needed as glucose-lowering therapies need to be de-escalated gradually. This will not only help to avoid hypoglycemia if glucose-lowering medication is initiated, but we will also be able to treat instances of persistent hyperglycemia more effectively. Furthermore, we need to repeat the glycemic assessment during follow-up and at the end of treatment so as to better guide the long-term care of TB survivors.

## Conclusions

Glycemic assessment needs to be performed in a setting of acute infection, as any new onset of hyperglycemia requires anti-glycemic drug therapy. The hyperglycemia presenting during TB infection may be stress hyperglycemia or overt diabetes; hence, it is necessary to closely monitor the blood glucose levels as well as drug therapy during the course of treatment for TB. Glucose-lowering therapies might need to be de-escalated gradually. Further, strict monitoring of blood glucose will also help in ensuring optimum treatment for overt diabetes in TB survivors. In the wake of the SARS-COVID-19 pandemic, this study had a reduced sample size at follow-up; hence, this study can be planned in a larger population with TB infection to understand the quantum of all states of hyperglycemia during and after the infection.
